# A Pilot Study Exploring the Use of Breath Analysis to Differentiate Healthy Cattle from Cattle Experimentally Infected with *Mycobacterium bovis*

**DOI:** 10.1371/journal.pone.0089280

**Published:** 2014-02-24

**Authors:** Christine K. Ellis, Randal S. Stahl, Pauline Nol, W. Ray Waters, Mitchell V. Palmer, Jack C. Rhyan, Kurt C. VerCauteren, Matthew McCollum, M. D. Salman

**Affiliations:** 1 Animal Population Health Institute, College of Veterinary Medicine and Biomedical Sciences, Colorado State University, Fort Collins, Colorado, United States of America; 2 United States Department of Agriculture, Animal Plant and Health Inspection Service, Wildlife Services, National Wildlife Research Center, Fort Collins, Colorado, United States of America; 3 United States Department of Agriculture, Animal Plant and Health Inspection Service, Wildlife Services, National Wildlife Research Center, Fort Collins, Colorado, United States of America; 4 United States Department of Agriculture, Animal Plant and Health Inspection Service, Veterinary Services, Wildlife Livestock Disease Investigations Team, Fort Collins, Colorado, United States of America; 5 United States Department of Agriculture, Agricultural Research Service, National Animal Disease Center, Ames, Iowa, United States of America; INIAV, I.P.- National Institute of Agriculture and Veterinary Research, Portugal

## Abstract

Bovine tuberculosis, caused by *Mycobacterium bovis*, is a zoonotic disease of international public health importance. Ante-mortem surveillance is essential for control; however, current surveillance tests are hampered by limitations affecting ease of use or quality of results. There is an emerging interest in human and veterinary medicine in diagnosing disease via identification of volatile organic compounds produced by pathogens and host-pathogen interactions. The objective of this pilot study was to explore application of existing human breath collection and analysis methodologies to cattle as a means to identify *M. bovis* infection through detection of unique volatile organic compounds or changes in the volatile organic compound profiles present in breath. Breath samples from 23 male Holstein calves (7 non-infected and 16 *M. bovis*-infected) were collected onto commercially available sorbent cartridges using a mask system at 90 days post-inoculation with *M. bovis*. Samples were analyzed using gas chromatography-mass spectrometry, and chromatographic data were analyzed using standard analytical chemical and metabolomic analyses, principle components analysis, and a linear discriminant algorithm. The findings provide proof of concept that breath-derived volatile organic compound analysis can be used to differentiate between healthy and *M. bovis*-infected cattle.

## Introduction

Bovine tuberculosis (bTB) is caused by *Mycobacterium bovis*, a zoonotic pathogen of importance to public health and international trade [1], [2]. Globally approximately 8.8 million incident cases of human tuberculosis occurred in 2010 [3], and while *M. tuberculosis* was responsible for the majority of those cases, an unknown proportion were likely attributable to *M. bovis* [4], [5]. Eradication programs and milk pasteurization have decreased the incidence of bTB in developed countries [6]; however, in developing countries, disease prevalence in cattle may approach 10–14% [7], [8]. Presently, in the United States of America (USA), ante-mortem surveillance tests for cattle include the caudal fold skin test (CFT), the comparative cervical skin test (CCT), and the interferon gamma assay (IFN-γ, IGRA; Bovigam, Prionics Ag, Schlieren-Zurich, Switzerland). While these tests have reasonable sensitivities and specificities [1], [6], [9], all take 48–72 hours to produce results, and require multiple animal handlings (CFT, CCT) or specialized laboratory procedures (IFN-γ). In addition, performance of these tests can be compromised by factors affecting the immune response or confounding test interpretation [10]. Other *in vitro* assays (i.e., serologic assays, lymphocyte proliferation assay, polymerase chain reaction) have limitations associated with their accuracy and execution relative to ante mortem surveillance [6].

There is emerging interest in diagnosing disease via identification of volatile organic compounds (VOCs) produced by pathogens, host-pathogen interactions, and biochemical pathways. Volatile organic compounds may be found in the blood, breath, feces, sweat, skin, urine, and vaginal fluids of humans and animals [11]–[13]. The suite of VOCs found in these samples is influenced by host biological variables such as age, breed, gender, genetics, metabolic function, and physiological status; environmental factors including diet, climate, husbandry, and seasonal variation; symbiotic and infectious microbe-host interactions; and pathophysiological responses to infections, toxins, or endogenous metabolic pathway perturbations [11], [14]. Volatile organic compound analysis has been used in human and veterinary medicine to explore suites of VOCs associated with infectious diseases [14]–[21], metabolic disorders and diseases [22]–[25], neoplasia [11], [26], [27], and organ transplant rejection [28]. Additionally, analysis of VOCs may prove useful for investigating metabolic and biosynthetic pathway processes associated with homeostasis and pathophysiological responses to disease. In cattle VOC analysis has been explored as a method for diagnosis of bovine respiratory disease [20], brucellosis [14], bovine tuberculosis [29], Johne's disease [14], [30], ketoacidosis [31], [32], and normal rumen physiology.

Studies searching for host-derived biomarkers of disease have classically been conducted using biofluids, cells, or tissues. Such biomarkers are likely present as well in expired air, since breath contains hundreds of endogenous and exogenous VOCs [33], [34]. To date, VOC analysis has been used to search for unique biomarkers associated with *M. bovis* and *M. tuberculosis* in serum samples [14], [35], [36], cell cultures [36]–[41], tissues [42], and breath [18], [29], [36]. Most research has attempted to isolate unique VOC biomarkers that would indicate presence of mycobacterial infection, with little work done to investigate potential changes within host VOC profiles that represent host-pathogen interactions or host responses to disease presence. Development of a highly sensitive and specific diagnostic tool capable of identifying such changes in VOC profiles would be of value in that sample collection would be non-invasive, easily repeatable, cost and labor efficient, and could be used in a point-of-care or “cow-side” setting. In this paper, we present the results of a pilot study exploring the concept of using VOC biomarkers in breath as a means to differentiate between non-infected cattle and cattle experimentally infected with *M. bovis*.

## Materials and Methods

### Ethics Statement

Strict biosafety level-3 (BSL-3) safety protocols were followed during all challenge and animal handling procedures to protect personnel from exposure to *M. bovis*. All animal work was reviewed and approved by the Institutional Biosafety and Animal Care and Use Committees (IACUC) of the United States Department of Agriculture (USDA), Agricultural Research Service (ARS), National Animal Disease Center (NADC), Ames, Iowa, USA; and the USDA, Animal and Plant Health Inspection Service (APHIS), National Wildlife Research Center (NWRC), Fort Collins, Colorado, USA prior to initiation of studies.

### Mycobacterium bovis challenge strains

Two strains of *M. bovis* were used for challenge inoculum: (1) *M. bovis* strain 95-1315 (USDA, APHIS designation) originally isolated from a white-tailed deer in Michigan, USA [43]; (2) *M. bovis* strain 10-7428_CO_Dairy_10-A (*M. bovis* strain 10-7428; USDA, APHIS designation) a recent isolate from Colorado, USA. Strains were prepared using standard procedures in Middlebrook 7H9 liquid media (Becton Dickinson, Franklin Lakes, NJ) [44].

### Animals and *Mycobacterium bovis* challenge

Male Holstein calves (n = 23, approximately 1 year of age) were obtained from a *M. bovis* and *M. avium paratuberculosis*-free herd in Wisconsin, USA, transported to NADC, and housed outdoors for approximately 2 months prior to placement into a BSL-3 agricultural facility at NADC. Animals were randomized to three treatment groups: non-infected controls (n = 7); animals receiving 10^4^ colony forming units (cfu) *M. bovis* strain 95-1315 (n = 8); animals receiving 10^4^ cfu *M. bovis* strain 10-7428 (n = 8) by aerosol as described by Palmer et al 2002 [45]. Each treatment group was housed according to IACUC guidelines in separate biocontainment rooms with no exchange of air, feed or water occurring between rooms. All animals were housed under the same environmental conditions, fed the same diet, and were allowed to acclimate to the new environment for approximately 3 months prior to initiation of *M. bovis* challenge studies.

### Diagnostic Tests Performed

Blood was collected from all calves at 2 weeks pre-challenge and at 2, 3, 4, 6, 8, and 12 weeks post-challenge for *in vitro* evaluation of cellular immune responses (CMI) to mycobacterial antigens including recombinant Early Secretory Antigenic Target -6kDa: Culture Filtrate Protein 10 fusion protein (rESAT-6:CFP10), overlapping (14 mer) peptide cocktail of ESAT-6:CFP10, *M. bovis* purified protein derivative (PPD), and *M. avium* PPD using the Bovigam assay [46]. Comparative cervical tuberculin (CCT) skin tests were performed at 12 weeks post-challenge as specified for the eradication of bovine tuberculosis in the United States [47]. All animals were humanely euthanized approximately 3.5 months after challenge by intravenous administration of sodium pentobarbital and necropsied. Tissues collected for bacteriologic isolation of *M. bovis* and histopathologic analysis included: parotid, medial retropharyngeal, mediastinal, and tracheobronchial lymph nodes; lung; and liver. Tissues were processed for isolation of *M. bovis*, and gross and microscopic lesions present were staged I–IV as previously described [45], [48].

### Collection of VOC samples

Breath sample collection was conducted 90 days post inoculation (DPI) and took place over three days, with one day dedicated to each treatment group (Day 1: control treatment group; Day 2: *M. bovis* strain 95-1315; Day 3: *M. bovis* strain 10-7428). Sampling commenced and concluded at the same time each day. Collection intervals per calf were approximately consistent for every animal in the study. A modified equine nebulization mask (Aeromask, Trudell Medical International, London, Ontario, Canada) was used for breath sample collection. Modifications included installment of three one-way valves to which charcoal filters were affixed to remove environmental VOCs from inspired air, installment of a one-way valve to allow excess expired air to escape, modification of the silicon gasket to allow proper fitting to the muzzle of the test subjects, and placement of a port at the apex of the mask to allow attachment of the breath sample kit. Breath sample kits consisted of: a 5 cm section of Tygon tubing (3/8 inch OD, ¼ inch ID) (Thermo Fisher Scientific, Inc., Waltham, MA, USA), a 3-piece bioaerosol cassette (SKC Inc., Eighty Four, PA, USA) containing a 37 mm 0.22 um PTFE filter (Tisch Scientific, North Bend, OH, USA) and a 37 mm cellulose pad (SKC Inc. Eighty Four, PA, USA); a 20 cm section of Tygon tubing; a Tenax sorbent cartridge (SKC Inc. Eighty Four, PA, USA); and a 20 cm section of Tygon tubing attached to a vacuum pump (AirChek XR5000, SKC Inc., Eighty Four, PA, USA). Each calf was restrained unhaltered in a standard cattle stanchion. The mask was held over the animal's muzzle and breath samples were collected at a rate of 1 L/min for 2 minutes (min). For background control, room air samples were collected three times during the duration of animal sampling each day using the same apparatus without the mask attached. Immediately post-collection, each Tenax cartridge was capped, placed in a Whirl-Pack (Nasco, Fort Atkinson, WI, USA) and stored at −80°C. Samples were transported on dry ice to NWRC, and stored at −80°C until analysis.

### Method Validation

To establish the working range of the gas chromatography/mass spectrometry (GC/MS) analysis method, 50 mg Tenax samples were spiked with 0.01 ml of a low (∼5 µg/mL) or high (∼250 µg/mL) alkane stock solution containing each of the following alkanes: decane (C10); undecane (C11); dodecane (C12); tridecane (C13); and tetradecane (C14). Samples were allowed to equilibrate following vortexing, at room temperature for 45 minutes. Samples were extracted in 0.5 mL hexane and analyzed by GC/MS using the same method for the breath samples to establish repeatability and limits of detection for the method. Linearity for the method was established across the range of 0.24–10.0 µg/mL for each of the alkanes. Spiked samples were replicated at n = 5 and the process was repeated on three separate occasions to allow for inter- and intra-day comparisons for method performance. Method limits of detection for each of the alkanes were calculated as a concentration that would produce a peak height three times the base line noise, measured peak to peak, based on the total ion current (TIC) chromatograms from the low fortified samples. Inter-day recoveries were evaluated based on the magnitude of the standard deviation as a percent of the target concentration (+/−20%), while intra-day recoveries were compared using ANOVA at α = 0.05.

### Sample Preparation for GC/MS Analysis and GC/MS Conditions

One Tenax sorbent cartridge from each animal was used for GC/MS analysis. A 50 mg sample of Tenax was extracted from each cartridge, and mixed with 0.5 mL hexane solvent. Each sample was sonicated for 10 minutes and the solvent then decanted into a GC vial. Analysis was performed using an Agilent 6890 GC coupled with an Agilent 5973 MS. Five microliters of sample solvent were injected into the GC in pulsed spitless mode. The inlet port temperature was 235°C, and the pulse pressure was 206.8 kPa (30 psi) for 0.5 minutes. The carrier gas was helium delivered with an average velocity of 59.0 cm/s. The column used was a DB-5 ms 30 m×250 µm column with a film thickness of 0.25 µm (J&W Scientific, Agilent Technologies, Santa Clara, CA, USA). Analytes were eluted from this column using a thermal gradient starting at 30°C and ramping at a rate of 5°C/1.0 min to a final temperature of 150°C. The total GC run time was 26.5 min. The temperature of the transfer line was 280°C. The MS was operated in positive ion mode, performing a total ion scan ranging from 10 to 550 m/z with a threshold of 150 m/z at a scan rate of 20 Hz. The MS source was operated at 230°C with the quad set to 150°C. Data were generated as raw Agilent.dat files.

### Data Processing

Data were analyzed qualitatively to identify VOCs present in the chromatograms, and quantitatively to determine if treatment group effects could be detected based on the ion abundances in the observed peaks. Chromatograms were baseline corrected using the region from 23–25 min, allowing for greater feature distinction in the chromatograms. Significant peak features in the chromatograms were identified using two different approaches. Initially features were identified using the Agilent Enhanced Chemstation MSD Data Analysis Tool software (Agilent Technologies, Santa Clara, CA, USA) and tentative peak compound identification was determined using the National Institute of Science and Technology (NIST) W8N08 database (www.nist.gov). Peaks were identified as significant if the total peak area exceeded 5000 across all ions in the peak. Compounds identified in the chromatograms using this approach were evaluated as possible metabolites using the Kyoto Encyclopedia of Genes and Genomes Database (KEGG) (www.genome.jp/kegg/) [49].

Chromatograms were also processed using XCMS Online (www.xcmsonline.scripps.edu) [49], [50] [51]. Briefly, this software identifies single ion (m/z) features that are significantly different across chromatograms grouped by treatment. Peaks identified in the chromatograms are aligned by a mean retention time calculated across all chromatograms evaluated in the data set. Peak features with relative intensity variance between sample groups are identified and a cross-sample peak-matching is performed in the METLIN Metabolite Database, identifying peaks that may represent metabolites [50], [52]. The ions identified in this analysis as significantly different across treatment groups were then used in the chemometric analysis described below.

### Statistical Analysis

Mass spectral data from the XCMS Online analyses were used to construct two sets of principle components analysis (PCA) and linear discriminant analysis (LDA) classification models using the chemometrics statistical package in “R” [53]. Initial PCAs were calculated using the data from the control and one *M. bovis* treatment group. The individual ions were median centered and scaled to a variance of 1.0 using the median absolute deviation. Outliers were identified as exceeding regular observations by the 97.5% quantile of a standard normal distribution of either distance value, and by visually plotting the score distance and the orthogonal distance against the sample number. Identified outliers were removed from subsequent analyses. Principle component analysis scores from *M. bovis* treatment groups were compared to the same control treatment group, and then used to parameterize LDA classification models using 2, 3, or 4 PCA scores [51].

The LDA classification models were written as two class models; classifying a sample as either a control or one of the *M. bovis* strains. A training dataset was constructed by randomly distributing two-thirds of the data, and a classification dataset was constructed from the remaining one-third of the data. The LDA classification was performed for 100 iterations and the resulting predicted classification of each test animal in a given iteration was compared to the actual treatment group assignment. Misclassification rates were calculated as a percentage of the total number of test animals misclassified per iteration of the model.

We compared the ability of our LDA classification models to correctly identify control *vs*. infected cattle to currently used surveillance tests by calculating diagnostic sensitivity and specificity using the PCA scores generated from the XCMS Online analysis. The best LDA classification model was used for each calculation (four PCA scores *M. bovis* strain 95-1315; three PCA scores *M. bovis* strain 10-7428). For both *M. bovis* strains, the numbers of true positive (*M. bovis*-infected) and true negative (control) samples classified across 100 iterations of the classification simulation were summed. Samples that were misclassified as falsely positive (negative sample incorrectly classified as positive) or falsely negative (positive sample incorrectly classified as negative) were also summed. Diagnostic sensitivity was calculated as the total number of true positives divided by the sum of the true positives plus false negatives. Diagnostic specificity was calculated as the sum of all true negative samples divided by sum of the true negative plus false positive samples [54]. These values are reported as percentages.

## Results

### Diagnostic Tests

Specific CMI responses of all calves prior to and during the study are reported elsewhere [46]. Briefly, prior to initiation of the study, in some calves, Bovigam assay results demonstrated responses to *M. avium* PPD that exceeded respective responses to *M. bovis* PPD indicating environmental exposure to ubiquitous non-tuberculous *Mycobacteria* spp. (NTM). During the study, the CMI responses of all *M. bovis*-inoculated calves to mycobacterial antigens were robust, with no significant differences noted between animals infected with *M. bovis* strain 95-1315 *vs*. *M. bovis* strain 10-7428. As early as three weeks post-challenge, CMI responses by all *M. bovis*-inoculated calves exceeded the pre- and post-challenge responses by the uninoculated controls. All calves inoculated with *M. bovis*, regardless of strain, were classified as reactors based upon standard interpretation of the CCT skin test 12 weeks after challenge. Calves in the non-infected control group were classified as negative on CCT skin test. During the study, no significant differences in clinical disease severity were observed between calves infected with *M. bovis* strain 95-1315 *vs*. *M. bovis* strain 10-7428. The severity of disease present grossly and microscopically was mild in both *M. bovis*-inoculated treatment groups. Similar gross and microscopic lesions were observed in the mediastinal and tracheobronchial lymph nodes and lungs of all *M. bovis*-inoculated calves examined (*M. V. Palmer, unpublished data*). *M. bovis* was isolated by culture from all calves inoculated with *M. bovis* strain 95-1315 or *M. bovis* strain 10-7428. *Mycobacterium bovis* was not isolated from the non-infected control group.

### Method validation

Method validation recoveries of low and high target concentrations for the alkanes were as follows: decane 0.55, and 5.5 µg/mL; undecane 0.52 and 5.2 µg/mL, dodecane 0.52 and 5.2 µg/mL, and for both tridecane and tetradecane 0.53 and 5.3 µg/mL. The retention times observed for each of the compounds were 8.9 minutes for decane; 12 minutes for undecane; 15.1 minutes for dodecane; 18 minutes for tridecane; and 20.8 minutes for tetradecane. The observed mean (mean +/−1 standard deviation) concentrations determined for each of the alkanes in the extracting solutions across the three repetitions of the procedure are presented in Table 1. All observed concentrations fell within 20% of the target. Standard deviations for the means fell within 10% of the mean. Limits of detection for each of the compounds across three repetitions of the extraction procedure consistently fell below 0.1 µg/mL. The concentrations of each of the alkanes observed across the three intraday repetitions of the procedure at the high and low fortification levels were not significantly different at the α = 0.05 significance level.

**Table 1:** Solvent extraction method development mean alkane concentrations observed across replicates.

Replicate/Alkane (ppm)	C10 (decane)	C11 (undecane)	C12 (dodecane)	C13 (tridecane)	C14 (tetradecane)
**Day 1**					
**Low Mean High Mean MLOD**	0.52+0.01 5.70+0.33 0.056	0.48+0.00 5.55+0.35 0.068	0.49+0.02 5.67+0.37 0.079	0.50+0.01 5.82+0.38 0.067	0.48+0.01 5.89+0.44 0.068
**Day 2**					
**Low Mean High Mean MLOD**	0.47+0.05 5.06+0.59 0.051	0.42+0.05 4.75+0.60 0.060	0.43+0.04 4.87+0.62 0.048	0.45 + 0.05 4.87 + 0.63 0.059	0.47+0.06 4.93+0.65 0.056
**Day 3**					
**Low Mean High Mean MLOD**	0.53+0.05 5.70+0.33 0.070	0.50+0.06 5.55+0.35 0.093	0.51+0.05 5.67+0.37 0.087	0.51 + 0.05 5.82 + 0.38 0.075	0.51+0.05 5.89+0.44 0.092
**ANOVA Results**					
**Low Fortification Comparison F_critical_ = 3.89**	Df = 2,12 F = 0.78 P = 0.49	Df = 2,12 F = 1.99 P = 0.18	Df = 2,12 F = 3.24 P = 0.07	Df = 2,12 F = 1.22 P = 0.33	Df = 2,12 F = 0.004 P = 0.995
**High Fortification Comparison**	Df = 2,12 F = 1.36 P = 0.29	Df = 2,12 F = 2.38 P = 0.13	Df = 2,12 F = 1.88 P = 0.20	Df = 2,12 F = 3.00 P = 0.088	Df = 2,12 F = 2.42 P = 0.13

### Compound identification

The peaks identified by the Agilent analysis were quantified and peak areas could be tentatively determined for 14 compounds (Table 2) using the NIST W8NO8 mass spectral library. The volatile compounds tentatively identified included acetals; alcohols; aldehydes; amines; hydrocarbons; ketones; an amino acid; a piperidine compound; and a pyrrolidine compound. Five compounds (4-hydroxy-4-methyl-2-pentanone, benzaldehyde, 1-ethyl-2-pyrrolidinone, α, α - dimethyl-benzenemethanol, and nonanal) were present in significantly greater concentration (p<0.05) in the *M. bovis*-infected treatment groups.

**Table 2:** Total Ion Chromatogram (TIC) peak area summary results of VOC profiles across treatment groups.

			Control		bTB strain 10-7428		bTB strain 95-1315		ANOVA (df 2, 19)	
Compound	Major m/z	Retention Time (min)	Mean	Standard Deviation	Mean	Standard Deviation	Mean	Standard Deviation	F statistic	p-value
1,1-diethoxyethane	45, 73,103	3.28	5013	3964	92881	148133	75599	179478	0.863	0.438
Toluene	91, 65, 39	3.78	15729	3106	11740	3284	13232	3190	2.930	0.078
Diethylamine	41, 56, 44	4.37	8363	5154	11084	4265	8756	7168	0.527	0.599
4-hydroxy-4-methyl-2-pentanone	43, 57, 58	5.46	8540	1491	23982	9902	18286	6603	8.905	1.87E-03
Styrene	104, 78, 51	6.37	21439	21783	17429	3627	15621	4002	0.390	0.682
Benzaldehyde	77, 106, 105,	8.31	13449	5213	15314	2932	9442	3312	3.606	0.047
1-ethenyl-2-pyrrolidinone	56, 111, 55	9.65	3838	4841	12382	5837	9649	7045	3.930	0.031
1-methyl-3-piperidinone	43, 84, 113	9.77	4294	4439	10136	7923	8690	5222	1.780	0.950
2-ethyl-1-hexanol	57, 43, 42,	10.44	54162	25339	52353	41487	47641	34600	0.066	1.937
α-acetophenone	105, 77, 51	11.50	18646	10814	15665	4136	12354	8959	1.011	0.383
α,α-dimethyl-benzenemethonol	43, 121, 77	12.18	13372	11406	33399	13490	23657	12264	4.813	0.020
3-heptanone	43, 57, 71	12.72	5820	13272	3361	3655	3366	3668	0.237	0. 792
Nonanal	57, 41, 56	12.87	29126	11982	72466	20903	51064	23526	9.210	0.002
1-1-dimethyl-2-(1-methylethyl) cyclopropane	151, 69, 41	21.78	49884	23201	54879	24983	57668	52478	0.038	0.963

### Cloud Plots

**Figure 1:**
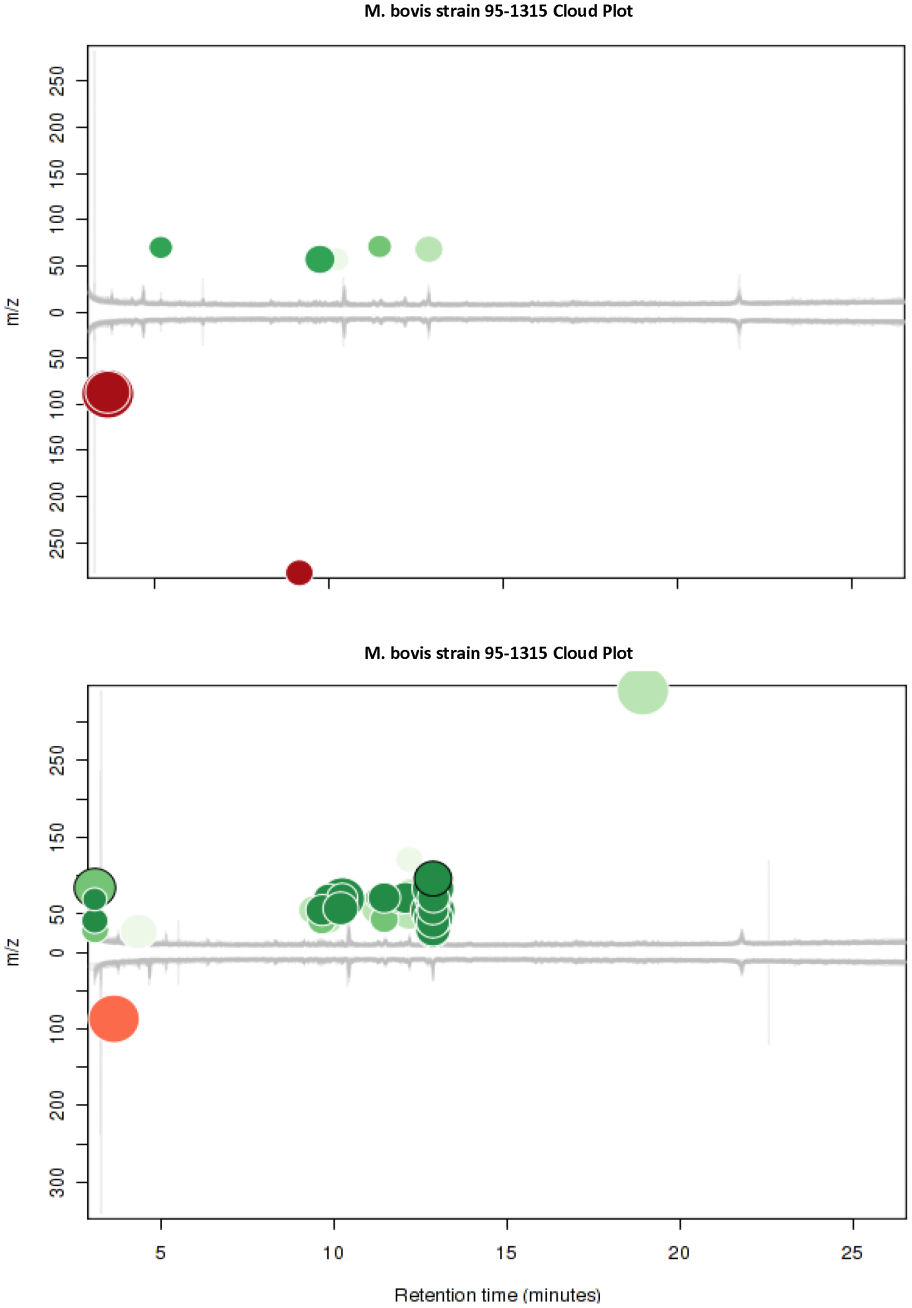
Cloud plots of aligned GC/MS chromatograms generated with XCMS Online. (**A**) Control vs. *M. bovis* strain 95-1315 analysis. (**B**) Control vs. *M. bovis* strain 10-7428 analysis. Control treatment group chromatograms are depicted below the X-axis, and *M. bovis*-infected chromatograms are positioned above. Up-regulated features of statistical significance are identified with green-colored circles located at the top of the plot, and down-regulated features are identified by red-colored circles located at the bottom of the plot. The color intensity of each circle represents the statistical significance of the feature difference, with brighter circles having lower p-values. The diameter of each circle represents a log-fold increase or decrease in abundance (i.e., larger circles correspond to peaks with greater fold differences).

The two aligned between-groups comparisons generated by XCMS Online are presented as cloud plots (Figure 1 A and B) [49], [55]. XCMS Online identified 137 peak features in the *M. bovis* strain 95-1315 *vs*. control group comparison, with 17 up-regulated features of statistical significance (p<0.05, >1.5 fold intensity change between treatment groups) present in the infected treatment group chromatograms (Figure 1A). There were 171 peak features identified in the *M. bovis* strain 10-7428 *vs*. control groups comparison with 51 features identified as significantly different between groups (p<0.01, >1.5 fold intensity change between treatment groups) (Figure 1B).

### Principle Components Analysis

**Figure 2:**
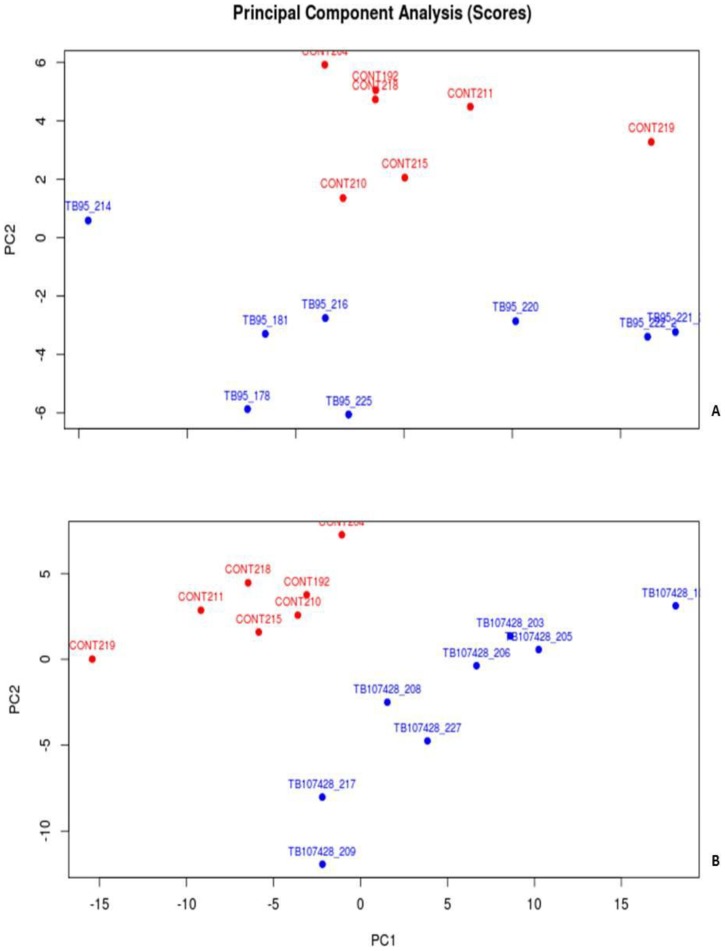
Principle Components Analysis results. (**A**) Control vs. *M. bovis* strain 95-1315. (**B**) Control vs. *M. bovis* strain 10-7428.

Principle components analysis plots were constructed using the first two principle components scores based on all the features identified in the XCMS Online analysis of the chromatograms. The ability to distinguish between *M. bovis*-infected and control group samples based on the spatial distribution of the treatment group scores is illustrated in Figure 2A and B. In both comparisons there is distinct clustering of treatment group samples and a well-defined separation between the infected group and control group sample clusters, indicating that the VOC profiles of the *M. bovis*-infected cattle are distinctly different from those of the control cattle. It is interesting to note that while the chromatograms of *M. bovis* strain 95-1315-infected cattle did not contain many statistically significant peaks (n = 17; p<0.05, >1.5 fold intensity change between treatment groups) (Figure 1A), the magnitude of peaks present did allow for differentiation, particularly after relaxing the 1.5 fold increase criteria and including ion fragments that met only the p<0.05 criteria.

### Linear Discriminant Analysis and Sensitivity and Specificity

Linear discriminant analysis models based on ions identified by XCMS Online as significantly different across treatment groups (p<0.01, *M. bovis* strain 10-7428; p<0.05, *M. bovis* strain 95-1315) did allow for classification (Table 3). The misclassification probabilities (combined false positive and false negative) for the control vs. *M. bovis* strain 10-7428 model were 11.25%, 8.75%, and 12.00%; and the misclassification probabilities for the control vs. *M. bovis* strain 95-1315 model were 22.09%, 17.50% and 2.25% (based on 2, 3, or 4 principle component scores, respectively). Based on the LDA model classifications, the sensitivity and specificity for the control vs. *M. bovis* strain 10-7428 and control vs. *M. bovis* strain 95-1315 were 83.8% and 96.4% (based on the three score model) and 97.4% and 99.2% (based on the 4 score model), respectively.

**Table 3:** Misclassification rates for Least Discriminant Analysis (LDA) models based on Principle Components Analysis (PCA) scores for XCMS Online data.

Number of PCA Scores Used in the Model	bTB strain 95-1315 *vs*. Control	bTB strain 10-7428 *vs*. Control
2	22.09%	11.25%
3	17.50%	8.75%
4	2.25%	12.00%
bTB (+) samples	n = 7	n = 7
Control samples	n = 8	n = 7
Number of variables	16	51
Training Data Set	n = 10	n = 10
Classification Data Set	n = 5	n = 4

## Discussion

In this pilot study we demonstrate that it is possible to discriminate between healthy cattle and cattle experimentally infected with *M. bovis* at 90 DPI, using GC/MS analysis of breath samples. The analytical and statistical approaches we describe provide a means of identifying compounds from breath analysis that may be diagnostically significant in identifying the presence of bovine tuberculosis infection. The cloud plots generated in the XCMS Online analysis demonstrate it is possible to differentiate between infected and healthy calves based on changes in ion intensities associated with VOCs common across the treatment groups. The results of our PCA further demonstrate this capability based on the distinct clustering of within group samples and the clear separation of between groups samples. The robustness of our models is supported by the low misclassification rates present in the LDA and by the calculated sensitivity and specificity values of our classification models, as those values observed compare favorably with the standard ante-mortem surveillance tests used in the United States [6], [9], [56], [57]. Our results were unexpected in that the intent of our work was to identify unique VOCs in the breath of *M. bovis*-infected cattle, based on the results of other studies exploring VOC analysis as a means of diagnosing tubercular disease in cattle [29] and other animal species [42], [58], with potential applications to humans [18], [36], [38], [39], [55]. However, our findings lead us to consider that the VOCs identified in our study represent up- or down-regulation of metabolic pathways, physiological or immune responses, or homeostatic perturbations caused by *M. bovis* infection.

The calves in our study were procured from a herd in which bovine tuberculosis- and *M. avium paratuberculosis*-infections were not reported or observed, were held in a controlled environment under observation for months prior to the start of and throughout the duration of the study, and were screened for exposure to *M. bovis*, *M. avium*, and *M. avium paratuberculosis* prior to challenge. In some animals, responses to *M. avium* PPD did exceed respective responses to *M. bovis* PPD prior to experimental infection with *M. bovis* indicating environmental exposure to ubiquitous NTM [46]. In general, NTM are rapidly cleared by cattle; thus, it was not anticipated that transient exposure and sensitization of the cattle to NTM would result in significant interference with detection and interpretation of *M. bovis* specific VOCs. The robust immune responses, gross pathologic and histopathologic observations, and bacteriological results in all *M. bovis* infected animals *vs*. controls lead us to state with confidence that the changes noted in the VOC profiles of the *M. bovis*-infected calves in our study were likely caused by *M. bovis* infection. We cannot, however, state that the changes noted in the breath VOC profiles are exclusive to *M. bovis* infection. Our findings do demonstrate that it is possible to differentiate between healthy and diseased calves, when *M. bovis* is present as the infectious agent. These results illustrate the need for further research exploring the breath VOC profiles of healthy cattle and those experiencing disease caused by *M. bovis* and other etiological agents in order to more thoroughly evaluate the robustness of VOC analysis as a disease detection method. To date, limited research has been conducted exploring the use of VOC analysis as a means to differentiate between healthy cattle and cattle infected with any etiological agent [19], [20], [29], [31], [32], [59]–[61]. This is likely due partly to the practical difficulties in adapting human breath sampling and analysis strategies to cattle, and in interpreting VOC profiles produced by animals that have a microbial fermentation-driven digestive system.

Tenax is widely used to concentrate nonpolar VOCs in air samples and is typically thermally desorbed before being analyzed by GC/MS [62]. However, Tenax has also been solvent extracted when used in air or aqueous phase sampling, particularly in environmental applications where large molecular weight compounds are being monitored [63]–[66]. The decision to use a solvent extraction method in this study was driven by the possibility that large organic molecules entrained in breath water vapor might be retained on the Tenax and would not be thermally labile. This is presently the subject of ongoing work.

We were able to provide tentative identification of 14 compounds using the Agilent Enhanced Chemstation MSD Data Analysis Tool/NIST W8N08 (Table 1). Seven of the compounds have been previously described in association with cattle [19], [29], [31], [32], [67]–[71], or as potential biomarkers for *M. bovis* [29], [40] or *M. tuberculosis* [41], [72] (Table 4). Tentative identification and change in peak intensity of nonanal is interesting as this compound is a lipid peroxidation by-product present in the breath of healthy humans and detected in greater concentrations in the breath of humans with respiratory tract disease [73], [74]. Potential metabolic pathway associations were identified for 6 compounds (toluene; styrene; benzaldehyde; 2-ethyl-1-hexanol; α-acetophenone; 1, 1-dimethyl 2-(1-methylethyl) cyclopropane)(Table 4). Review of the literature identified one other study exploring the VOC profiles of cattle infected with *M. bovis*. In that study 16 VOCs were tentatively identified, with 10 VOCs present in the breath of all the cattle sampled, four VOCs apparently exclusive to healthy cattle, and two VOCs apparently exclusive to cattle infected with *M. bovis*. Only two VOCs were consistent between that study and our study. Acetophenone was found in the breath of all cattle in both studies. Nonanal was present in the breath of all cattle in our study, but was absent from the breath VOC profiles of *M. bovis*-infected cattle and present only in a subset of healthy cattle in the other study [29].

**Table 4:** Comparison of compounds identified in cattle and humans.

Compound	Cattle	Humans	Culture	Potential metabolic pathway [78]	Other [71], [79]
1,1-Diethoxyethane					Found in onions, grapes. Used as a flavoring ingredient in fruit and alcohols. Endogenous metabolite. Food metabolite.
Toluene	Ketosis [31], [32] BRD [19] *M. bovis* [29]			*M. tuberculosis M. bovis* BCG *Bos tarus*	Found in allspice, lime oil and some foods. Food metabolite. Toxin and pollutant metabolite. Found in some plants.
Diethylamine	Healthy [70]				Occurs naturally in some foods and plants. Endogenous metabolite.
4-Hydroxy-4-methyl-2-pentanone			M. tuberculosis [41]		Also known as diacetone alcohol. Found in fruits. Endogenous metabolite. Food metabolite.
Styrene	Healthy [67]	Tuberculosis [72]		*M. tuberculosis M. bovis* BCG *Bos tarus*	Found naturally in some plants and a variety of foods including fruits, vegtables, nuts, beverages, meats and dairy products. Exhibits signaling and catabolic functions. Food metabolite. Biofunctions include catabolism and signaling.
Benzaldehyde				*M. tuberculosis M. bovis* BCG *Bos tarus*	Occasionally found as a volatile compound in urine. Food additive. By-product in phenylalanine metabolism.
1-Ethenyl-2-pyrrolidinone					Also known as polyvidone. Used as a food additive. 2-pyrrolidinone is a lactam cyclization product of gamma-aminobutyric acid (GABA). Food metabolite.
1-Methyl-3-piperidinone					
2-Ethyl-1-hexanol	Healthy [69]			*M. tuberculosis M. bovis* BCG *Bos tarus*	May occur naturally in some fruits and grains, olive oil, tobacco, and teas. Endogenous metabolite. Food metabolite. Biofunctions include cell signaling, energy source, and membrane integrity.
α-Acetophenone	Healthy [69] BRD [19] *M. bovis* [29]			*M. tuberculosis M. bovis* BCG *Bos tarus*	Found in some plants. Used as a food flavoring ingredient. Additive in cigarettes. Has anti-fungal properties. Drug metabolite. Food metabolite.
α,α-Dimethyl-benzenemethanol					
3-Heptanone					Found naturally in spearmint. Used as a flavoring ingredient. Endogenous metabolite. Food metabolite.
Nonanal	BRD [19] *M. bovis* [29]	Tuberculosis [72] Asthma, COPD [73], [74]		*M. tuberculosi M. bovis* BCG *Bos tarus*	Lipid peroxidation by-product
1-1-Dimethyl-2-(1-methylethyl) cyclopropane					Cyclopropane fatty acids are produced by some microorganisms and plants. American Oil Chemists Society (AOCS) Lipid Library www.lipidlibrary.aocs.org

While it is conceivable that some VOCs produced by monogastric animals, humans, cattle, and bacteria may be similar, there is limited continuity in the suites of VOCs identified when comparing studies performed on healthy cattle *vs*. cattle with BRD or *M. bovis* infection, studies of healthy humans *vs*. humans with tuberculosis, and between *M. tuberculosis* cultures grown *in vitro* in different types of solid and liquid media [18]–[20], [29], [31], [32], [41], [59], [61], [67], [68], [72], [75]. Volatile compounds identified as biomarkers for specific pathogens in culture or preliminary human or animal testing have been found in the breath of normal subjects and subjects with diseases of different etiology, or associated with specific foods or other materials [41], [73], [76]. Likely explanations for these inconsistencies include individual variability; similarities in host response to pathogen presence; pathobiological similarities between pathogens; endogenous and exogenous factors; and, relative to cattle, the dynamic nature of rumen gases. Identifying endogenous and exogenous factors that may affect VOC suite composition and concentrations of VOCs present in breath is important. Endogenous VOCs are comprised of blood-borne compounds produced by metabolic, hemostatic, or pathologic processes that passively diffuse across the blood-alveolar interface or are produced within the respiratory tract. Exogenous VOCs present in the environment that are passively inspired then expired, or are present in food and water may be inadvertent contaminants [76].

The diverse methods of VOC collection and analytical methods that have been used are likely to have contributed to the variability in results as well. For example, methods of sample collection have included Tedlar bags, Tenax sorbent cartridges, and SPME fibers of various types [19], [20], [29], [35], [60], [61], [67], [75], and sample analysis methods have included, but have not been limited to, thermal desorption-GC/MS [19], [61], [67], proton-transfer-reaction mass spectrometry (PTR-MS)[75], electronic nose technology coupled with GC/MS [35], [60], nanotechnology based artificial nose (NA-NOSE) in combination with GC/MS [29], in addition to our solvent sample extraction-GC/MS method. The methods of VOC identification when performed have been variable as well.

Our study demonstrates the importance of analysis method and database selection for purposes of compound identification. The Agilent Enhanced Chemstation MSD Data Analysis Tool/NIST W8N08 search emphasized identification of unique chromatographic features, whereas the XCMS Online/METLIN search focused upon identification of feature differences between groups, with emphasis on minor peaks within chromatograms. Standard chemical databases such as NIST W8N08 contain many classes of compounds including industrial solvents, toxicants, and biohazardous materials. Metabolomic database searches appear more likely sources for identification of compounds produced by living organisms or cell-based structures; however, the number of compounds and species represented in metabolomics databases are often limited [49], [77]. Utilizing a combined chemometric-bioinformatics approach may provide the best method for identification of unique or dysregulated peaks within chromatograms until such time that metabolomic databases are capable of functioning as standalone references.

The potential influence of endogenous and exogenous VOCs, the variability in collection strategy, analysis methodology, and VOC identification underscores the difficulty of identification of VOCs as biomarkers for specific pathogens or diseases, and the need for cross-validation and standardization of breath analysis methods. It will be especially important to consider the potential confounding influences of endogenous and exogenous VOC sources when performing breath analysis on animals under field conditions. In principle, breath analysis could be applicable to all animal species, although modification of systems used in human breath analysis is required. In many animal species sample collection is not voluntary and collection of an alveolar breath sample is not possible. This necessitates capture of breath samples via mask or nasal collection systems [61], and expectations that samples will likely contain VOCs derived from the upper respiratory or gastrointestinal tracts.

The strengths of this study include the ability to control for many endogenous and exogenous factors that might affect breath VOC profiles. The test subjects were all male Holstein calves of the same approximate age housed under the controlled environmental and dietary conditions. Inoculum preparation and the nebulization method used were consistent. Sample collection was conducted over the same time period on consecutive days, and sample handling was consistent across all treatment groups. Limitations of this study include the low number of study animals, immunological evidence of prior exposure to NTM in some of the test subjects, and lack of comparative breath analysis research in healthy cattle, tuberculous cattle, or cattle infected with BRD or other etiological agents.

Continued investigation and refinement of our breath collection system and our methods may lead to development of diagnostic strategies and disease surveillance monitoring systems that could preclude individual animal handling. Advantages to such systems would include decreased stress on individual animals, decreased cost and labor, ability to screen groups of animals, and potential surveillance of wildlife reservoirs of zoonoses and diseases of agricultural importance. Future work should include continued research using experimentally infected cattle and naturally infected cattle, multiple time point sample collections, collection of biofluid and tissue samples, increased sample sizes, comparative studies examining the VOC profiles produced by cattle with other infectious diseases and by cattle housed in different environments and fed different diets, and compound confirmation using reference standards. The eventual transfer of developed laboratory methods to portable GC-MS or Electric Nose systems would be beneficial and future work will ideally incorporate such tools.
